# Susceptibility of Protein Methionine Oxidation in Response to Hydrogen Peroxide Treatment–Ex Vivo versus In Vitro: A Computational Insight

**DOI:** 10.3390/antiox9100987

**Published:** 2020-10-13

**Authors:** Juan C. Aledo, Pablo Aledo

**Affiliations:** Departamento de Biología Molecular y Bioquímica, Facultad de Ciencias, Universidad de Málaga, 29071 Málaga, Spain; 0619874997@uma.es

**Keywords:** methionine sulfoxide, oxidative stress, protein oxidation, redox signaling

## Abstract

Methionine oxidation plays a relevant role in cell signaling. Recently, we built a database containing thousands of proteins identified as sulfoxidation targets. Using this resource, we have now developed a computational approach aimed at characterizing the oxidation of human methionyl residues. We found that proteins oxidized in both cell-free preparations (in vitro) and inside living cells (ex vivo) were enriched in methionines and intrinsically disordered regions. However, proteins oxidized ex vivo tended to be larger and less abundant than those oxidized in vitro. Another distinctive feature was their subcellular localizations. Thus, nuclear and mitochondrial proteins were preferentially oxidized ex vivo but not in vitro. The nodes corresponding with ex vivo and in vitro oxidized proteins in a network based on gene ontology terms showed an assortative mixing suggesting that ex vivo oxidized proteins shared among them molecular functions and biological processes. This was further supported by the observation that proteins from the ex vivo set were co-regulated more often than expected by chance. We also investigated the sequence environment of oxidation sites. Glutamate and aspartate were overrepresented in these environments regardless the group. In contrast, tyrosine, tryptophan and histidine were clearly avoided but only in the environments of the ex vivo sites. A hypothetical mechanism of methionine oxidation accounts for these observations presented.

## 1. Introduction

The accumulation of molecular oxygen in the primitive atmosphere opened the way for the harnessing of highly exergonic reactions based on O_2_ as a terminal electron acceptor, leading to an efficient aerobic metabolism able to support larger sized forms of life [1]. The importance of oxygen in multicellular organisms’ development is not limited to its bioenergetic role as a final electron acceptor in mitochondrial respiration. Thus, molecular oxygen is involved in a myriad of biosynthetic pathways, many of which are essential for specialized cell functions found exclusively in multicellular organisms [2]. However, all of these metabolic innovations did not come without a price paid in terms of oxidative stress. During aerobic metabolism, a small and variable amount of the consumed oxygen is converted to reactive oxygen species (ROS). In the past, ROS production was thought of as a dangerous and unavoidable by-product of aerobic metabolism. However, now we know that mitochondrial ROS, although potentially harmful when in excess, actually plays important roles in various redox-dependent signaling processes [3]. Thus, cells have evolved mechanisms to sense the redox status and transduce that information to trigger adequate responses. In this context, the hypothesis that the oxidative modification of methionine residues may participate in cellular signaling has gained attention during the last years [4].

Methionine can be oxidized to methionine sulfoxide (MetO) by a varied list of oxidizing agents [5,6]. Upon oxidation, the sulfur atom becomes a new stereogenic center and two diasteroisomers, Met-S-O and Met-R-O, can be formed. These sulfoxides can be reduced back to methionine in a reaction catalyzed by ubiquitous methionine sulfoxide reductases (Msrs) [7]. Canonical MsrA and MsrB have been shown to be absolutely stereospecific for the reduction of Met-S-O and Met-R-O, respectively [8]. More recently, a new enzymatic system, MsrQ/MsrP, which can reduce both R- and S-diasteroisomers has been described in gram-negative bacteria [9]. Thus, like phosphorylation, methionine oxidation is a reversible covalent modification that has been demonstrated to both up regulate [10,11,12] and down regulate [13,14] protein activity through the direct sulfoxidation of specific methionine residues. Furthermore, methionine oxidation can also impact on protein activity indirectly by coupling oxidative signals to protein phosphorylation-dephosphorylation [15,16]. On the other hand, there is abundant evidence that the dysregulation of this methionine oxidation-reduction cycle could be the basis of multiple diseases [17]. Not surprisingly, the interest in characterizing methionine oxidation has been revived and a number of proteome-wide studies of methionine oxidation has been reported in the past few years [18,19,20,21,22,23,24]. In an additional effort to identify, classify and document sulfoxidized proteins/sites in different organisms and under different experimental conditions, we have recently built a database, MetOSite, that provides easy access to information on experimentally confirmed sulfoxidized methionine sites [25].

Herein, we have taken advantage of the wealth of data provided by MetOSite to comparatively assess the similarities and differences between MetO-containing proteins that have been oxidized inside living cells (ex vivo) with respect to those MetO-containing proteins that were oxidized in cell-free preparations (in vitro). To this end, we collected data coming from proteomic studies where human cell lines were exposed to hydrogen peroxide and then the proteins were extracted and analyzed by mass spectrometry (ex vivo dataset). On the other hand, data from proteomic studies in which cell-free extracts were oxidized with hydrogen peroxide and then analyzed, formed the in vitro dataset In a first approach we adopted a protein-centric perspective; that is, we analyzed the properties of the MetO-containing proteins. Afterward, we moved the focus from the global protein properties to the local sequence environment of MetO sites. By combining these two approaches, we aimed to find distinctive features that may help, in the long term, to understand when a given methionyl residue may be signaling-competent.

## 2. Materials and Methods

### 2.1. Data Collection

MetOSite (https://metosite.uma.es) is a database devoted to integrating resources for the study of methionine residues sulfoxidation [25]. Thus, we wrote an R script to programmatically interact with the MetOSite’s API (application programming interface) in order to query the database for all of the sites that matched with human proteins that have been described to contain methionine sulfoxide after treatment with hydrogen peroxide. Both the script (metosite.R) and the processed dataset can be downloaded from https://bitbucket.org/jcaledo/characterization-of-human-meto-sites. As MetOSite provides information regarding the experimental conditions—that is, whether the oxidation reaction took place ex vivo or in vitro—we split the data into two disjoint sets: set A, formed by 1113 proteins containing 1904 MetO sites described to be oxidized ex vivo and set B consisting of 503 proteins containing 1994 MetO sites described to be oxidized in vitro. A table providing the bibliographic references relating to the proteomic studies used to assemble these datasets can be found as Supplementary Material (Table S1). It should be noted that, with the exception of the von Willebrand factor (P04275), all of the proteins analyzed in the current paper were detected as MetO-containing proteins during the course of proteomic studies; that is, they were not recombinant proteins.

### 2.2. Intrinsically Disordered Regions and Protein Abundances

To compute the fraction of proteins containing disordered regions, the UniProt ID of each single protein from the set under analysis was used to query the database DisProt [26]. To this end, we wrote an ad hoc script (disprot.R) to programmatically interact with the DisProt’s API, which is accessible at https://bitbucket.org/jcaledo/characterization-of-human-meto-sites. The protein abundance data were obtained from the database resource PaxDB, a comprehensive absolute protein abundance database [27]. This information can be found as part of the provided dataset where the abundance variable is expressed as parts per million.

### 2.3. Empirical Null Distributions

To assess the statistical significance of the observed methionine frequency, protein length, protein abundance and the content of intrinsically disordered regions in proteins from sets A and B empirical null distributions were generated as described below. One hundred thousand samples, each including 1113 human proteins, were randomly collected from UniProt. For each sample, the mean and median of methionine frequency and protein length were computed as well as the mean protein abundance and the fraction of protein of the sample that contained intrinsically disordered regions (IDR). These random distributions allowed us to evaluate the statistical significance of the corresponding values measured in set A (ex vivo). On the other hand, to evaluate the significance of the values observed for proteins from set B (in vitro), a similar process was carried out but in this occasion each of the 100,000 samples was formed by 503 proteins randomly taken from the whole human proteome.

### 2.4. Analysis of Subcellular Distribution of MetO-Containing Proteins

We considered ten potential subcellular compartments that are indicated in Table 1. For each protein analyzed, we searched for the presence (coded as 1) or absence (coded as 0) of the corresponding cellular component gene ontology (GO) terms. In this way, a 10-dimensional vector was used to encode the cluster to which the protein belonged. In other words, we defined an equivalence relation where two proteins were related if their 10-dimensional vectors were the same. For instance, according to Table 1, a protein present only in the nucleus would exhibit a vector such as (0, 1, 0, 0, 0, 0, 0, 0, 0, 0). All of the proteins showing such a vector would belong to the equivalence class (cluster) “nucleus”. As a given protein can populate different compartments, the maximal theoretical number of clusters that can be formed is 2^10^. However, only 133 out of the 1025 theoretically possible clusters were found in our dataset; most of them containing only a few proteins. Thus, for further analysis, only those clusters containing at least 10 proteins were considered (Table S2). 

To address whether in vitro and ex vivo oxidized proteins showed a differential enrichment within these clusters, a Fisher’s exact test was carried out. To this end, a contingency table was built as follows. In the entry of the table corresponding to the first row and the first column, the number of proteins oxidized ex vivo found in the analyzed cluster was annotated. The entry of row 1 and column 2 accounted for the number of proteins oxidized in vitro found in the cluster. On the other hand, the second row and first column gave the number of proteins oxidized ex vivo found in all of the clusters other than the one being analyzed, while the second row and second column collected the number of proteins oxidized in vitro found in clusters other than that under analysis. For instance, when analyzing the cluster of proteins grouped into the nucleus, we obtain Table 2.

In this concrete example, we can conclude that the cluster “Nucleus” is significantly enriched (*p*-value < 2.2 × 10^−16^) with proteins from the set “Ex Vivo”.

### 2.5. Gene Ontology Protein Network Analyses

To formalize the analyses, we began by defining the sets *P* and *O*:

P=A ∪B

O :={all the GO terms of human proteins}

where *A* and *B* are the ex vivo and in vitro sets, respectively. Each element from *P* is identified by its corresponding UniProt ID. Next, we defined the function *f* as follows:

 f:P → P(O)

p ⟼f(p)={GO terms annotated to protein p}

where P(O) is the powerset of *O*; that is, the set formed by all of the subsets of *O*. In this way, we are ready to define an endorelation over the set *P*. For this purpose, we will say that $p_{i}$ and $p_{j}$ are related, which is denoted as $p_{i}\;R\;p_{j}$, if and only if $p_{i}$ and $p_{j}$ share at least 50% of its GO terms; that is, if their Jaccard similarity index is equal or greater than 0.5:

(1) $$\frac{\left|f\left(p_{i}\right)\cap f\left(p_{j}\right)\right|}{\left|f\left(p_{i}\right)\cup f\left(p_{j}\right)\right|}\;\geq 0.5.$$

Formally, a graph G(V, E) is a mathematical structure consisting of a set of vertices, *V*, and a set of edges, *E*, where its elements are unordered pairs, (a,b)∈E, of distinct vertices, a, b ∈V. In our case, we built and analyzed the following graph:

$$G=\left(V=P,\;E=\left\{\left(p_{i},\;p_{j}\right)\in P\;x\;P\;:\;p_{i}\;R\;p_{j}\right\}\right).$$

The code used to compute the Jaccard and adjacency matrices as well as to build and plot the graphs can be obtained at https://bitbucket.org/jcaledo/characterization-of-human-meto-sites/src/master/GO_network.Rmd.

### 2.6. Assortativity

The network described above includes vertices of two types corresponding to proteins oxidized either ex vivo or in vitro. When vertices of a type show a trend to associate with others that are like them, we say that the network shows assortative mixing. If, on the contrary, the vertices prefer to associate with others that are of a different type, the network is said to show disassortative mixing. To quantify the level of assortative mixing in our network, we computed the assortativity coefficient described by Newman [28], which is implemented in the R package igraph [29]. Briefly, a type mixing matrix, $\left(e_{ij}\right)$, can be defined where the element $e_{ij}$ is the fraction of edges in the network connecting a vertex of type *i* to one of type *j*. As we are dealing with undirected graphs, this matrix is symmetric; that is, $e_{ij}=\;e_{ji}$. This matrix satisfies the sum rules $\sum_{ij}e_{ij}=1,\;\sum_{j}e_{ij}=\;a_{i},\;\sum_{i}e_{ij}=b_{j}$, where $a_{i}$ can be interpreted as the probability of finding an edge leaving a vertex of type *i*, while $b_{j}$ can be seen as the probability of finding an edge reaching a vertex of type *j*. On an undirected unipartite graph, as it was in our case, $a_{i}=b_{i}$. If there is no assortative mixing, then $e_{ij}=a_{i}b_{j}$. If there is assortative mixing, this can be measured by calculating the standard Pearson correlation coefficient:

(2) $$r=\frac{\sum_{ij}ij\left(e_{ij}-a_{i}b_{j}\right)}{\sigma_{a}\sigma_{b}}$$

where $\sigma_{a}$ and $\sigma_{b}$ are the standard deviation of the distributions of $a_{i}$ and $b_{j}$. The value of *r* lies in the range −1 ≤r≤1 with *r* = −1 indicating perfect disassortativity; that is, a perfect negative correlation between *i* and *j*. On the other hand, *r* = 1 indicates perfect assortativity.

### 2.7. Co-Regulated Protein Pairs Analysis

In a recent work, Kustatscher et al. identified 62,812 co-regulated human protein pairs [30]. We selected those co-regulated protein pairs where both members of the pair belonged to the set A∪B, where *A* was the set of protein being exclusively oxidized ex vivo and *B* was the set of protein being oxidized exclusively in vitro. This selection procedure resulted in 5530 protein pairs of which 2723 pairs were formed entirely by proteins from set *A* (pure ex vivo pairs), 1726 pairs contained a member from set *A* and the other member from set *B* and 1081 pairs contained exclusively proteins from set *B*. As our interest was to address whether proteins from set *A* appeared together forming pairs more often than we might expect when all of the potential pairs are equiprobable (there is no selectivity) we defined a random variable, *F*, as the fraction of pure ex vivo pairs in a sample of 5530 taken from a population formed by all of the potential pairs.

As the co-regulated pairs are not ordered pairs—that is, (protein X, protein Y) is the same pair as (protein Y, protein X)—the number of all potential pairs formed with our MetO-containing dataset was $\frac{N\left(N-1\right)}{2}$ where *N* = 1616 (total number of proteins). On the other hand, the number of possible ex vivo pure pairs in this population would be $\frac{N_{vv}\left(N_{vv}-1\right)}{2}$ where *N_vv_* = 1113 (the number of proteins in the ex vivo set). Therefore, under null hypothesis conditions (there is no selectivity), the expected proportion of pure ex vivo pairs was $E\left[F\right]=\frac{N_{vv}\left(N_{vv}-1\right)}{N\left(N-1\right)}$. We then simulated an empirical probability distribution of the random variable *F*. For this purpose, from the population of all of the potential pairs, we randomly took a sample of 5530 pairs. Each pair could either be or not a pure ex vivo pair with probability E[F] and 1−E[F], respectively. The fraction of pure ex vivo pairs obtained in this sample was recorded. This sampling procedure was repeated 100,000 times to generate the empirical probability distribution of *F*.

The raw data of co-regulated protein pairs as provided by Kustatscher and co-workers (corregulated_pairs.Rda) as well as the code (corregulation.R) used to carry out the above described analyses can be obtained from https://bitbucket.org/jcaledo/characterization-of-human-meto-sites/src/master.

### 2.8. MetO Sites Sequence Environments

For each MetO site the frequency of the flanking amino acids at each position from −10 to +10 relative to the central MetO was recorded. Only methionyl residues with ten or more neighbors in both directions were included in this analysis. Thus, we obtained two square matrices of order 20, $\left(f_{ij}^{MetO\_vivo}\right)$ and $\left(f_{ij}^{MetO\_vitro}\right)$ where $f_{ij}^{MetO\_vivo}$ was the relative frequency for amino acid *i* at position *j* computed in proteins oxidized exclusively ex vivo whereas $f_{ij}^{MetO\_vitro}$ was the relative frequency for amino acid *i* at position *j* computed now in proteins oxidized exclusively in vitro. For each oxidation site, a non-oxidized methionine residue within the same protein was randomly chosen and subjected to the same frequency analysis to derive the corresponding control frequency matrices, $\left(f_{ij}^{Met\_vivo}\right)$ and $\left(f_{ij}^{Met\_vitro}\right)$. Those proteins containing a higher number of MetO sites than non-oxidized Met sites were disregarded.

As we were interested in detecting the differences between the sequence environments of MetO sites and their control Met sites, we formulated the following null hypothesis. The difference between the relative frequency matrices of MetO sites and their Met control sites yielded the zero matrix. To contrast this hypothesis, two new matrices $\left(Z_{ij}^{vivo}\right)$ and $\left(Z_{ij}^{vitro}\right)$, accounting for the standardized differences in frequencies, were computed according to the following equation:

(3) $$Z_{ij}=\frac{f_{ij}^{MetO}-f_{ij}^{Met}}{\sqrt{\frac{f_{ij}^{MetO}\left(1-f_{ij}^{MetO}\right)+f_{ij}^{Met}\left(1-f_{ij}^{Met}\right)}{n}}}$$

where *n* is the total number of oxidation sites being analyzed in each case. Under the null hypothesis condition, this typified variable must follow a normal distribution with mean 0 and variance 1. That is, $Z_{ij}^{vivo}↝N\left(0,1\right)$ and $Z_{ij}^{vitro}↝N\left(0,1\right)$. Thus, standard scores $Z_{ij}$ that were significantly greater than or significantly less than 0 indicated a preference or disfavor, respectively, of the residue *i* at the position *j* around the MetO site.

## 3. Results and Discussion

**Figure 1:**
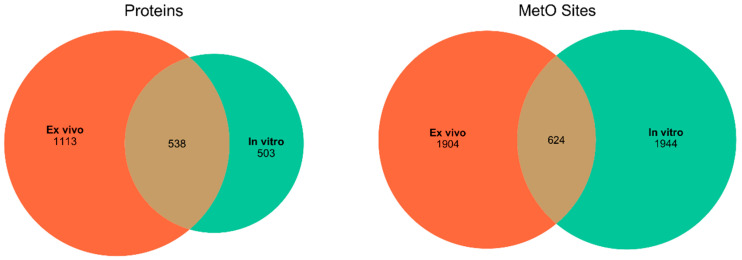
Distribution of the number of proteins and MetO sites found in ex vivo and in vitro studies. Left panel: distribution of the number of different MetO-containing proteins. Right panel: distribution of the number of MetO sites.

We started searching the MetOSite database to identify sites detected as methionine sulfoxide in human proteins after treatment with hydrogen peroxide either in vitro or ex vivo. After removing those methionines corresponding to initiation codons, a total of 4472 MetO sites belonging to 2154 different human proteins were identified and used in subsequent analyses. Around 43% of the MetO sites were reported to be oxidized exclusively in vitro; the other 43% were detected as MetO only in ex vivo experiments. The remaining 14% of the oxidized sites were detected in both in vitro and ex vivo studies. While the number of methionine sites oxidized ex vivo was roughly the same as those oxidized in vitro, the latter seemed to be concentrated in a smaller number of different proteins (Figure 1). In the following, we will distinguish between those results derived from a protein-centric approach and those derived from a local approach focused on MetO sites.

### 3.1. MetO-Containing Proteins Are Enriched in Methionine Residues and Intrinsically Disordered Regions

**Figure 2:**
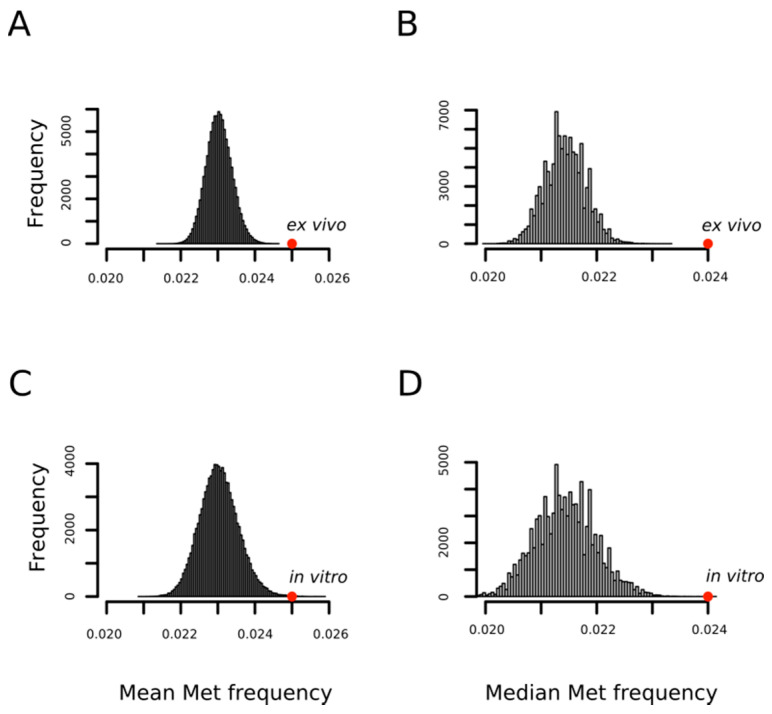
MetO-containing proteins are enriched in methionine residues. Empirical distributions of mean and median methionine content for 100,000 random samples from the human proteome. For each sample, both the mean (**A**,**C**) and the median (**B**,**D**) of the relative frequency of methionine were obtained by averaging across either 1113 (**A**,**B**) or 503 (**C**,**D**) proteins randomly chosen from the human proteome. The positions of the mean and median values computed for the set of 1113 proteins oxidized ex vivo (**A**,**B**) and for the set of 503 proteins oxidized in vitro (**C**,**D**) are indicated with the red circles.

In a previous work, using a limited set of oxidized proteins, we found that proteins detected as containing MetO were enriched in methionyl residues [31]. Now, we confirm and extend that result with the observation that the methionine content of a protein influences the probability of its oxidation regardless of the conditions under which the oxidation reaction takes place; that is, in vitro or ex vivo. Figure 2 shows how the mean frequencies of methionine among proteins being oxidized either ex vivo (0.025 ± 0.01) or in vitro (0.025 ± 0.01) are significantly higher than that expected by chance when proteins are randomly drawn from the human proteome to form sets of the same cardinality (*p*-values < 10^−4^ and = 7 × 10^−4^, respectively). The statistical significance was also high (*p*-values < 10^−4^) when medians were considered rather than means as measures of central tendency (Figure 2B,D).

**Figure 3:**
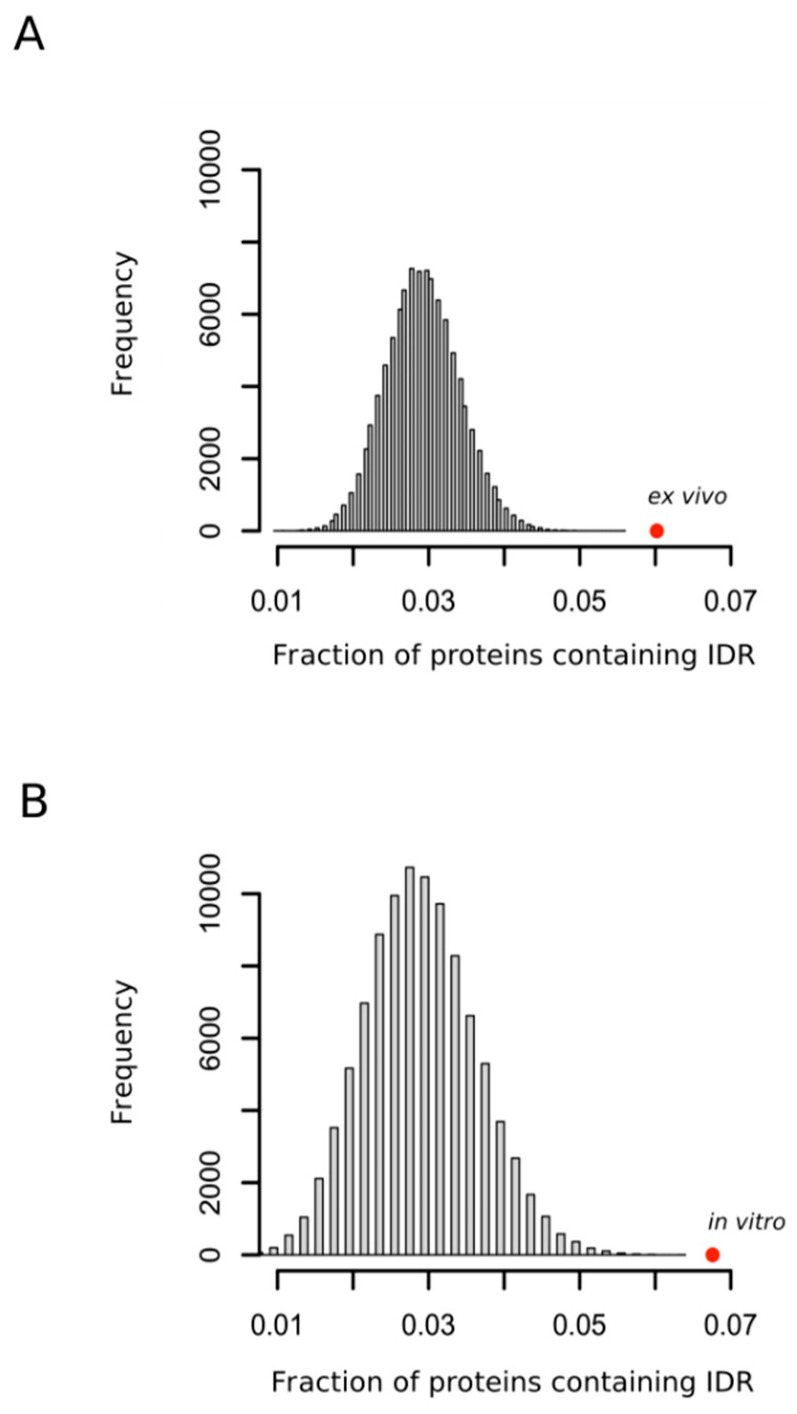
Oxidation prone proteins are enriched in protein containing intrinsically disordered regions. The fraction of proteins containing intrinsically disordered regions (IDR) in each random sample was computed. Each sample was formed by either 1113 (**A**) or 503 (**B**) proteins. In each case 100,000 samples were randomly taken from the whole human proteome. The fraction of proteins containing IDR observed for the sets of proteins oxidized ex vivo and in vitro were calculated and indicated as red circles (**A**,**B**), respectively.

Although the structural properties of a protein that modulates the reactivity of their methionyl residues have remained unclear [32] and the methionine residues that are oxidized are not always simply related to their accessibility to the external medium [33], the accessibility of the sulfur atom to solvent seems to be, nevertheless, an important factor of the propensity of that methionine to be oxidized [32,34]. Thus, in a recent work where protein extracts were oxidized in vitro with hydrogen peroxide, Walker and coworkers concluded that methionines within regions classified as disordered were significantly more prone to oxidation than those classified as structured [35]. Our analyses confirmed this observation (Figure 3A) and, more importantly, extended the conclusion to the case of protein oxidation taking place within the living cells (Figure 3B) where the presence of interacting partners and molecular chaperones may, in theory, impose structure on regions that otherwise appear disordered in vitro. 

### 3.2. Proteins Oxidized Ex Vivo Are Bigger and Less Abundant Than Proteins Oxidized In Vitro

In the above, we have described that proteins prone to methionine oxidation present a bias towards high methionine frequency and a trend to contain intrinsically disordered regions. These two features are shared by proteins oxidized ex vivo as well as proteins oxidized in vitro. Next, we will present another two features, protein size and protein abundance, whose distribution between these two categories of oxidized proteins is quite different.

The protein sequence length within a given proteome varies substantially from a few to thousands of amino acids. A statistical investigation of protein length distribution in proteomes reveals that evolution to higher forms of life imposes an increase in the mean and median protein length [36] suggesting that the evolution of eukaryotic proteins was influenced by processes of fusion of single-function proteins into extended multifunctional and multi-domain proteins. Thus, larger proteins can combine multiple domain structures in complex arrangements to increase enzyme specificity, provide links between other domains or regulate functional activity [37]. 

**Figure 4:**
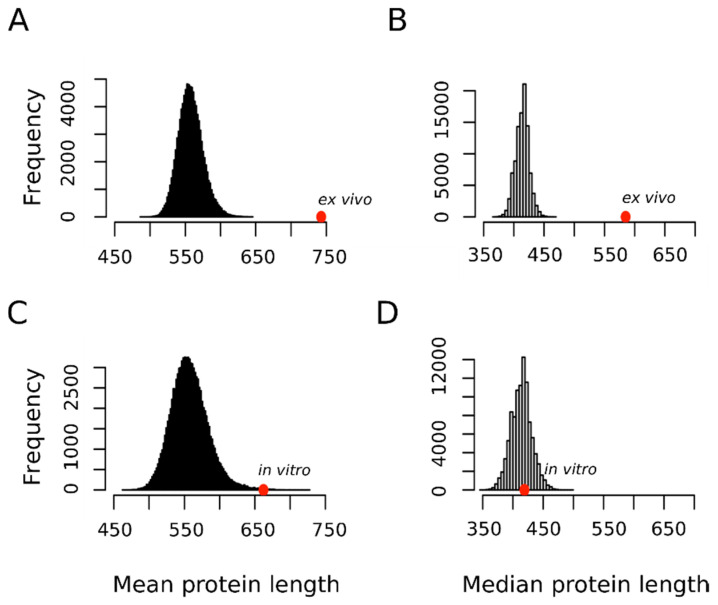
Mean and median protein length distribution. Empirical distributions of mean and median protein length for 100,000 random samples from the human proteome. For each sample, both the mean (**A**,**C**) and the median (**B**,**D**) protein length were obtained by averaging across either 1113 (**A**,**B**) or 503 (**C**,**D**) proteins randomly chosen from the human proteome. The positions of the mean and median values computed for the set of 1113 proteins oxidized ex vivo (**A**,**B**) and for the set of 503 proteins oxidized in vitro (**C**,**D**) are indicated with the red circles.

In this context, we hypothesized that if the oxidation of a not negligible fraction of the methionine residues sulfoxidized ex vivo represented a bona fide regulatory modification, then perhaps this fact may be reflected in the protein size distribution of this subset. Figure 4 shows that in contrast to proteins oxidized in vitro that did not depart from the expected distribution for the human proteome, the proteins oxidized ex vivo tended to be much larger than expected by chance. Thus, the mean ± standard deviation for the protein length in the ex vivo and in vitro sets were 742.8 ± 629.3 and 662.2 ± 1685.7 residue long, respectively. In none of the 100,000 random samples did we observe a mean as extreme as that for the ex vivo set (*p*-value < 10^−4^). However, in 169 random samples we observed a mean equal or greater than that computed for the in vitro set (*p*-value = 0.00169). This last *p*-value may tempt us to conclude that, although marginally significant, proteins oxidized in vitro tend to be larger than the average human protein. However, it should be noted that while the standard deviation for the protein size in the ex vivo set was below its mean, in the case of the in vitro set, the standard deviation was almost three times greater than its mean. As noted by other authors, when it comes to protein size, the median gives us a much better and more reliable information about our datasets mainly because of the presence of outliers [38]. This is particularly relevant in our case where titin, with its 33,423 residue long polypeptide, was the champion of the outliers and was an element of our in vitro set (https://metosite.uma.es/scan/Q8WZ42). Thus, when the median distribution was used instead of the mean distribution (Figure 4B,D vs. A,C), a clear-cut conclusion could be reached: a large protein size is a hallmark of proteins oxidized ex vivo but not in vitro.

**Figure 5:**
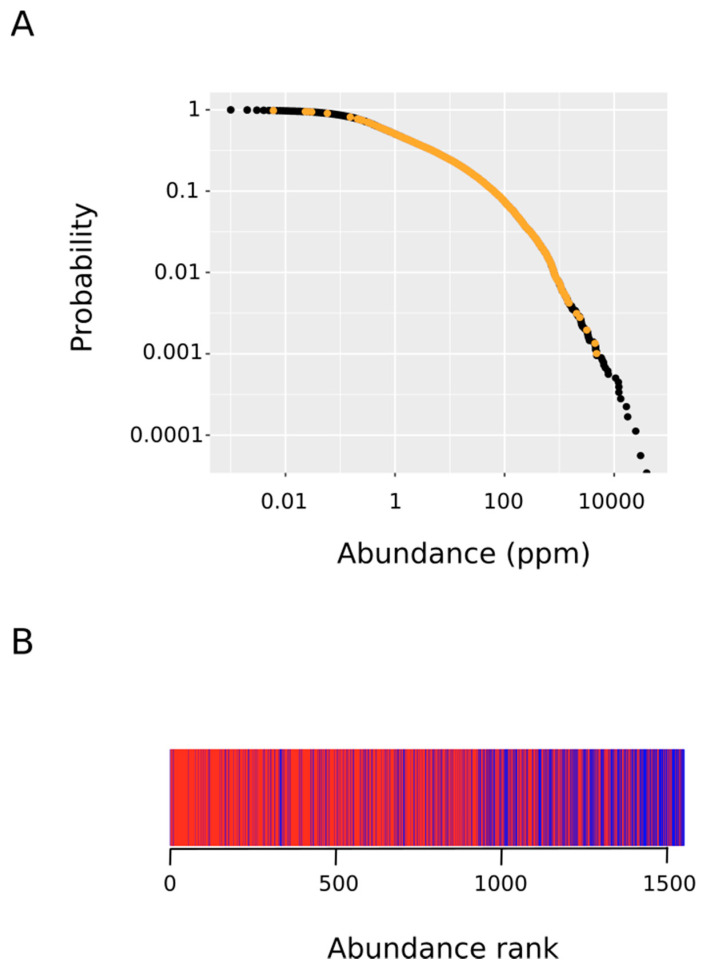
Rank-frequency plot of protein abundance. We started with a list of all of the human proteins along with their frequency of occurrence (abundance in parts per million). The complementary cumulative distribution P(x) of abundance x was defined as the fraction of proteins with abundance greater than or equal to x. (**A**) The plot depicts the observed complementary cumulative distribution (ordinate) versus protein abundance (abscissa). The points corresponding to MetO-containing proteins are indicated in orange. (**B**) The MetO-containing proteins were ranked in increasing order according to their abundances. Those rank positions occupied by a protein from the ex vivo set are marked in red while rank positions where a protein from the in vitro set was present are in blue.

Recent technological advances in the field of mass spectrometry have allowed for the systematic quantification of the absolute abundances of thousands of proteins [27]. This wealth of data enables insights into fundamental cellular biology. Thus, it has been established that steady-state protein abundance of orthologues is well-conserved across large evolutionary distances [39,40]. Therefore, protein abundance regulation mirrors specific biological roles and the steady-state abundances of proteins are thought to be determined by their functions [41]. Herein, we reasoned that if methionine sulfoxidation taking place within living cells in response to oxidative stress targets regulatory proteins as hypothesized, then their abundances should be below the average abundance for proteins oxidized in vitro where the chemical oxidation process, being less specific, should be mass-action driven. We started comparing the abundance distribution within the oxidized protein set with that for the whole human proteome (Figure 5A). As it can be observed, the abundance of the oxidized proteins differed over several orders of magnitude ranging from 0.006 to 4771 parts per million. Next, to test the hypothesis that proteins from the ex vivo set are significantly less abundant than their in vitro counterpart, we performed a Wilcoxon–Mann–Whitney test. To this end, we ranked in increasing order all of the MetO-containing proteins according to their abundances. In Figure 5B, those rank positions occupied by a protein from the ex vivo set are marked in red while rank positions where a protein from the in vitro set was present are in blue. This figure indicates that the first rank positions (low protein abundance) were dominated by protein oxidized ex vivo while the opposite end of the rank was heavily populated by proteins from the in vitro set. The Wilcoxon–Mann–Whitney test confirmed that these differences were highly significant (*p*-value < 2.2 × 10^−16^). 

### 3.3. Nuclear and Mitochondrial Proteins Are Preferentially Oxidized Ex Vivo

**Figure 6:**
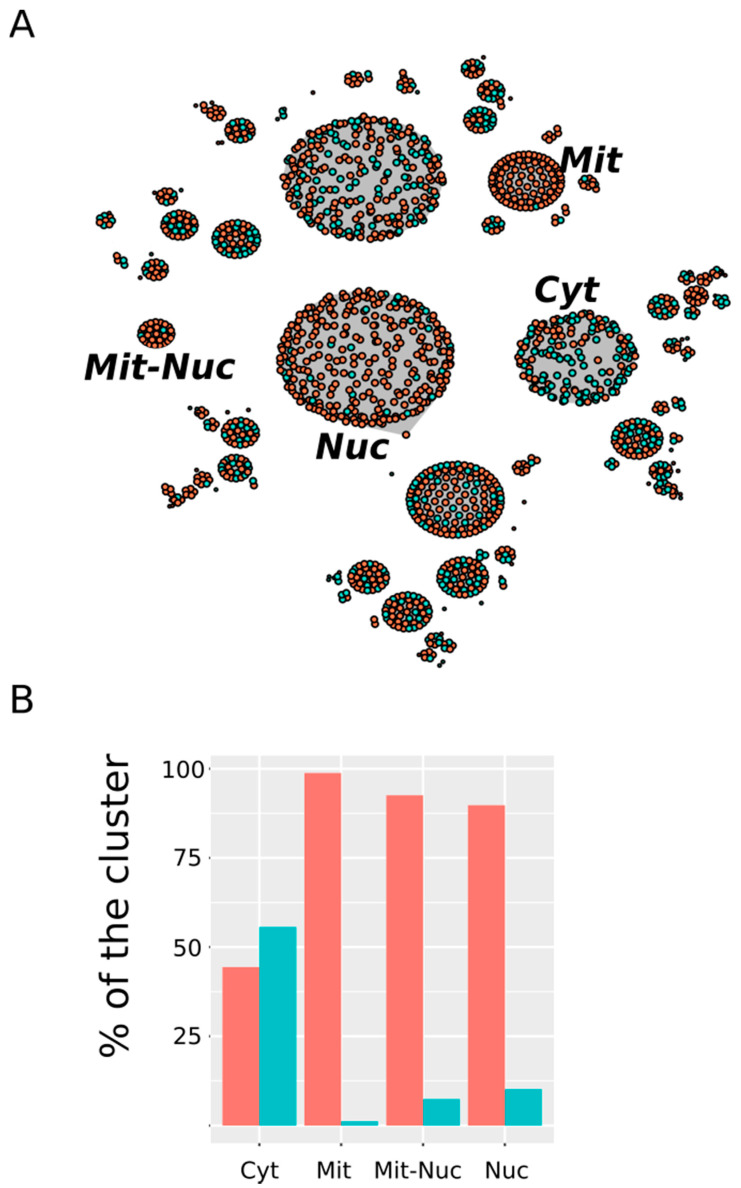
Subcellular distribution of MetO-containing proteins. (**A**) Network where each cluster represents an equivalence class containing proteins showing the same subcellular localization. Proteins from the ex vivo set have been represented in orange while protein belonging to the in vitro set are represented in turquoise. Only those clusters that show a significant (*p*-value < 0.005) preference for proteins from one of these two sets are labeled (Cyt: cytosol; Nuc: nucleus; Mit: mitochondrion; Mit-Nuc: mitochondrion-nucleus). (**B**) The percentages of the total number of proteins found in a cluster contributed by ex vivo (orange) and in vitro (turquoise) vertices are shown.

Another characteristic that we found to be distinctive of the subset of proteins being considered was the subcellular localization of the protein. In the current study, we considered ten potential subcellular compartments (see Methods) but because a given protein can be related to different compartments, up to 2^10^ different clusters can be formed; at least in theory. However, as expected, only a small fraction of all of these theoretical clusters was represented in our data (Figure 6 and Table S2). 

In Figure 6 we have highlighted those clusters showing a highly significant selectivity either for proteins from the in vitro set or for proteins from the ex vivo set. Among the first—that is, clusters enriched with in vitro oxidized proteins—only the cytosol (Cyt) stood out. Thus, while proteins oxidized in vitro represented only around 31% of all of the proteins in our dataset, they contributed nearly 56% to the cluster “cytosol” (*p*-value = 1.7 × 10^−6^). On the other hand, compartments related to the nucleus and the mitochondrion showed a clear preference for protein from the ex vivo set. Thus, around 90% of the protein grouped into the “nucleus” (Nuc) cluster belonged to the ex vivo set (*p*-value < 2.2 × 10^−16^). Similarly, 98.8% and 92.6% of the protein from the “mitochondrion” (Mit) and “mitochondrion-nucleus” (Mit-Nuc) clusters belonged to the ex vivo set (*p*-values = 3 × 10^−12^ and 5 × 10^−3^, respectively). In summary, whereas in vitro oxidized proteins were mainly cytosolic proteins, those proteins exclusively oxidized ex vivo were often related to the nucleus and the mitochondria.

### 3.4. The Ex Vivo and In Vitro Sets Exhibit Assortative Mixing in GO Protein Networks

When focusing on proteins, a natural question that arises is whether the proteins belonging to these two sets (proteins exclusively oxidized ex vivo, set A, and proteins exclusively oxidized in vitro, set B) share characteristics proper and distinctive of each set other than the features we have described above (protein size, protein abundance and protein localization). In other words, features related to biological processes and molecular functions. To address this question, we resorted to the mathematical concept of binary relation. The idea was to define an endorelation over the whole set of human proteins (see Methods for details) and then assess whether or not proteins belonging to set A were more often related among them than they were related to proteins from set B. To this end, we defined a relation based on the extent of GO terms in common between two proteins. Thus, when two proteins shared at least 50% of their GO terms we said that they were related.

Once we introduced a binary relation, we were in condition to take full advantage of the well-developed network theory to answer our question: are proteins from set A more often related among them than they are related to proteins from set B?

**Figure 7:**
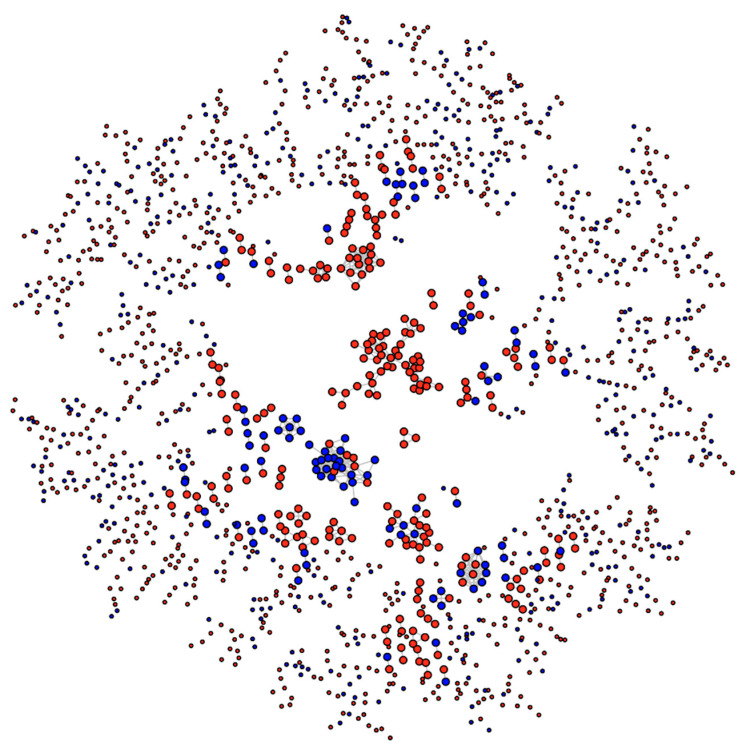
Network of human MetO-containing proteins. Two given proteins (nodes) are connected (linked by an edge) if they share at least 50% of their gene ontology terms. Nodes corresponding to proteins oxidized ex vivo are in red while those corresponding to proteins oxidized in vitro are in blue.

Consider Figure 7, which shows the network of proteins containing MetO sites either exclusively oxidized ex vivo (red nodes) or either exclusively oxidized in vitro (blue nodes). The tendency for nodes of one type to associate with nodes of the same type is called homophily or assortative mixing and can be quantified using the so-called assortativity coefficient, r, [28]. In general, for a network containing two types of nodes, the assortativity coefficient can range between −1, when a network is perfectly disassortative (every edge connects two nodes of different types) and 1 when there is perfect assortative mixing (every edge connects two nodes of the same type). When the link between two nodes is completely independent of the node’s type, the r takes values close to zero. For the network shown in Figure 7, we found a value for the assortativity coefficient of r = 0.53, indicating strong assortativity mixing. Nevertheless, to evaluate the significance of this result, we assessed the assortativity coefficient of 10,000 randomly mixed networks having otherwise the same network topological quantities (see https://bitbucket.org/jcaledo/characterization-of-human-meto-sites/src/master/GO_network.Rmd).

**Figure 8:**
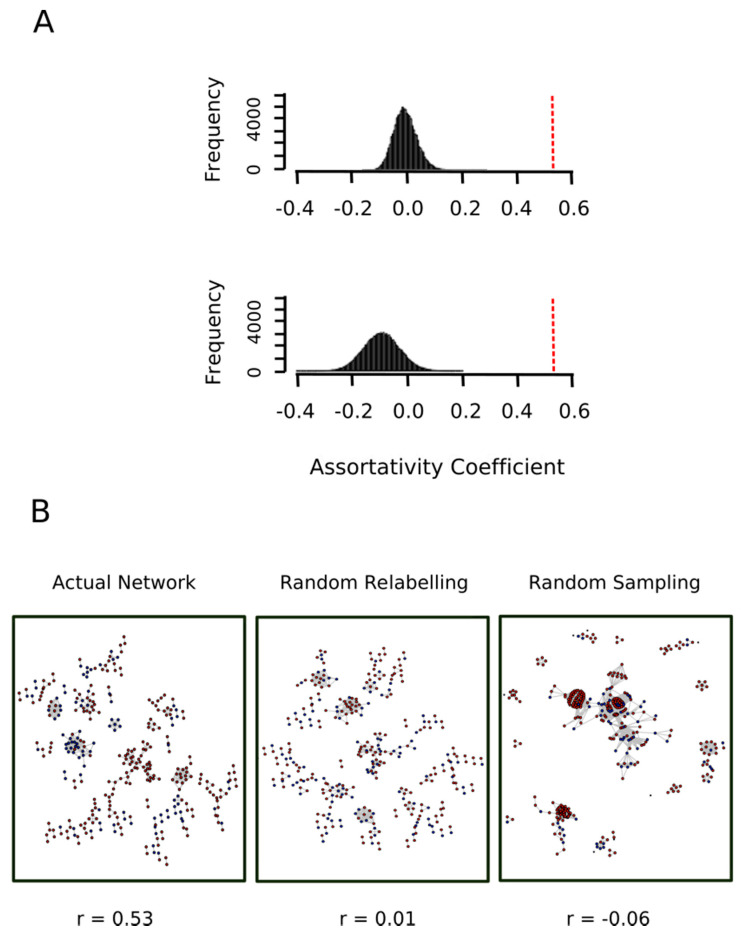
Proteins oxidized ex vivo and in vitro show a significant assortative mixing. (**A**) The nodes in the actual network shown in Figure 7 were randomly relabeled either as a red node or a blue node then the assortativity coefficient of the relabeled network was computed. This process was repeated 10,000 times and the assortativity coefficient distribution was plotted (top plot in panel A). On the other hand, the whole human proteome network was sampled to obtain 10,000 random subnetworks containing as many nodes as the actual network shown in Figure 7. For each of these subnetworks, 1113 nodes were randomly labeled as “red” and the remaining 503 nodes were labeled as “blue” and its assortativity coefficient was computed. The bottom plot in panel A shows the distribution of the assortativity coefficient in this random sampling experiment. The vertical red dashed lines indicated the value of the assortativity coefficient for the actual network shown in Figure 7. (**B**) The actual network and representative networks of the random relabeling and random sampling experiments are shown in B after removing isolated nodes (nodes with degree 0).

To this end, we kept the network shown in Figure 7 and each node was randomly re-labeled either as a red node or a blue node. The assortativity coefficient of the relabeled network was then computed. This process was repeated 10,000 times and the assortativity coefficient distribution was plotted. As expected, we obtained a normal distribution centered at r = 0 (Figure 8A, top panel), which allowed us to conclude that the value r = 0.53 observed for the actual network indicated a strong and highly significant (*p*-value < 10^−4^) assortative mixing.

To further strengthen this conclusion, we carried out a second control test where the topology of the network was not kept constant. In other words, we put forward the null hypothesis that the two sets (proteins exclusively oxidized ex vivo, set A, and protein exclusively oxidized in vitro, set B) represented random samples from the human proteome and therefore any relationship between their proteins would be merely random. To test this null hypothesis, the whole human proteome network was sampled to obtain 10,000 random subnetworks containing as many nodes as the actual network shown in Figure 7. For each of these subnetworks, 1113 nodes (cardinal of set A) were randomly labeled as “red” and the remaining 503 nodes (cardinal of set B) were labeled as “blue”. The bottom panel of Figure 8A shows the distribution of the assortativity coefficient for these random networks. As it can be observed, the distribution was centered at a slightly negative value. One might ask what this negative value signified. Why do we not simply have a distribution centered at r = 0? The answer is that a randomly mixed network is normally closer to a perfectly disassortative network than to a perfectly assortative network [28]. More interestingly, this empirical distribution allows us to reject the null hypothesis (*p*-value < 10^−4^). Thus, the assortative mixing among proteins oxidized ex vivo and protein oxidized in vitro cannot be explained by a random sampling effect.

### 3.5. Proteins Oxidized Ex Vivo Are More Often Co-Regulated Than Expected by Chance

In a recent work, Kustatscher and co-workers determined changes of abundance of human proteins in response to 294 biological perturbations using isotope-labelling mass spectrometry. These perturbations covered a wide range of biological conditions including perturbations with drugs and growth factors, genetic perturbations, cell differentiation studies and comparisons of cancer cell lines [30]. In this way, they were able to produce a co-regulation map of the human proteome, which is publicly available [30]. More concretely, these authors identified 62,812 protein pairs that behaved in a highly correlated way throughout most of the perturbation experiments. Herein, we reasoned that if proteins being oxidized ex vivo in response to hydrogen peroxide functioned together, then they may have similar patterns of up and down regulation. To address this question, we took advantage of the above referred co-regulation map of the human proteome. Thus, we started by filtering the co-regulated protein pairs to keep only those pairs in which the two members were MetO-containing proteins. In this way, the co-regulation map of human MetO-containing proteins boiled down to 5530 protein pairs of which 2723 pairs were formed entirely by proteins oxidized ex vivo; that is, pairs of co-regulated proteins where both members belonged to the ex vivo set.

**Figure 9:**
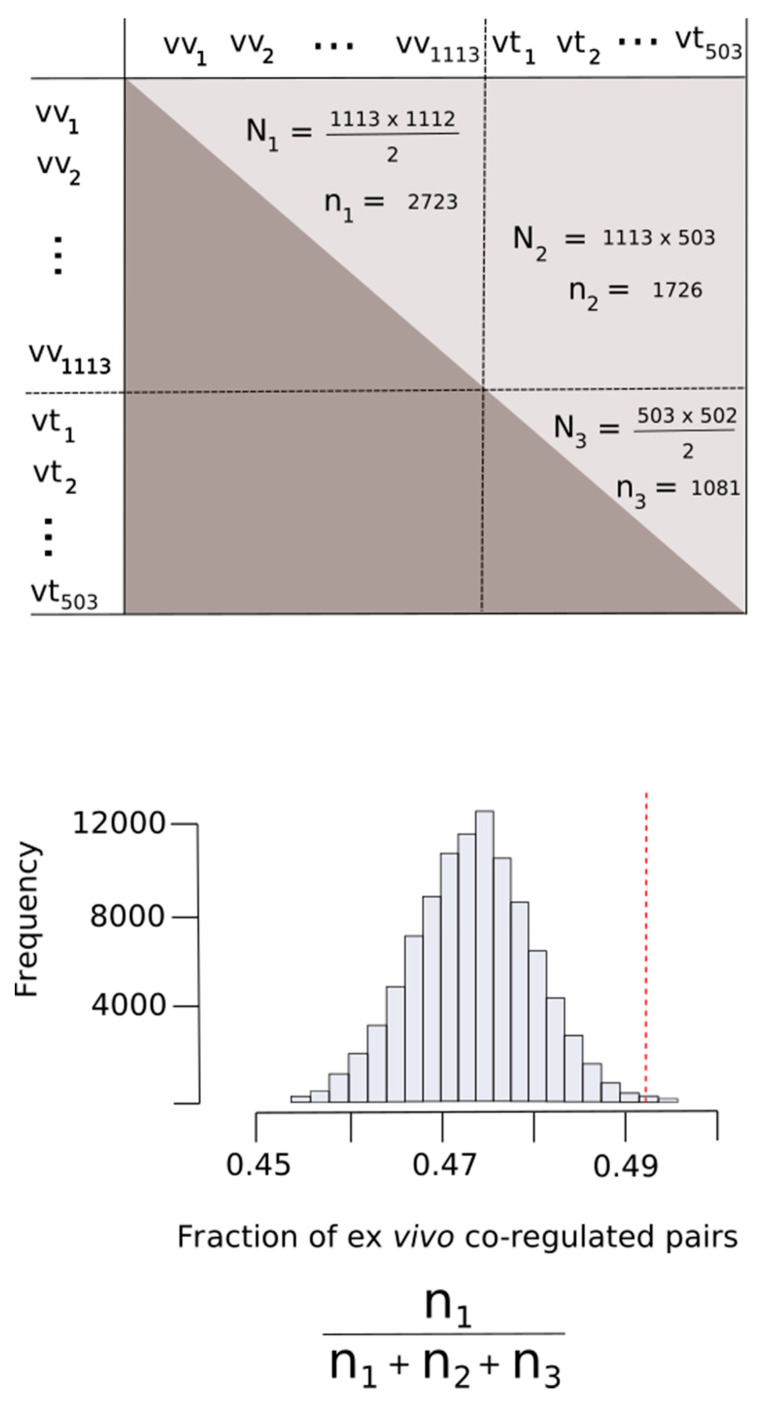
Proteins oxidized ex vivo are co-regulated more often than expected by chance. All of the potential pairs of proteins oxidized ex vivo (vv) and in vitro (vt) were considered. As the pair formed by a given protein with itself has no meaning in our context, the main diagonal of the matrix corresponding to this cartesian product was disregarded (upper panel). Given that the pair (x, y) is the same as the pair (y, x), our matrix is an upper triangular matrix. N_1_, N_2_ and N_3_ represent the number of elements from this matrix formed by pure ex vivo (vv, vv), mixed (vv, vt) and pure in vitro (vt, vt) pairs, respectively. On the other hand, n_1_, n_2_ and n_3_ correspond to the numbers of these pairs observed in our dataset. In the bottom panel is the distribution of the fraction of ex vivo co-regulated pairs expected when the pairs were sampled randomly. The vertical red dashed line indicated the actual fraction of the ex vivo co-regulated pairs observed.

In order to assess whether this observed fraction (2723/5530 = 0.492) was significantly higher with respect to the value that could be expected when all of the potential pairs were equiprobable (null hypothesis), we defined a random variable, *F*, as the fraction of pure ex vivo pairs obtained from a random sampling of 5530 pairs taken from the entire population of potential pairs (Figure 9). Under the null hypothesis conditions, the expected value of *F* was calculated to be E[*F*] = 0.474 (see Methods for details). The probability of obtaining a value of *F* greater or equal to the observed value of 0.492 was then assessed by taking 100,000 random samples of 5530 pairs for each sample. Figure 9 shows the empirical distribution of *F*, which allowed us to compute P[*F* ≥ 0.492] = 0.00315. Therefore, we rejected the null hypothesis and concluded that the number of times that proteins from the ex vivo set appeared as co-regulated pairs was greater than would be expected by chance (Figure 9). 

### 3.6. The Oxidation and Reduction of Methionine Residues Are Sequence Dependent

In previous studies using proteomic data derived from H_2_O_2_-stressed Jurkat cells, it was noticed that acidic amino acids closely surrounded the oxidized methionine (MetO) whereas tyrosine, tryptophan and histidine were underrepresented in the neighborhood of MetO [18,31]. The observation that acidic residues are frequently found in the vicinity of MetO sites has also been reported in the human carcinoma A431 cell line where methionines were photooxidized within living cells pre-treated with Photofrin [21]. Interestingly, when we analyzed the data provided by a proteome-wide study of in vivo methionine oxidation in Arabidopsis plants subjected to high light irradiation [20], we again observed that both glutamate and aspartate were significantly overrepresented in the neighborhood of MetO (Figure S1).

However, because in all of these proteomic studies the oxidation of methionine residues took place within living cells, the presence of MetO-containing proteins represents a steady-state situation in which oxidation, a chemical event, is balanced by reduction, an enzymatic process mediated by methionine sulfoxide reductases (Msrs), active in the living cell. In other words, the interpretation of the results of these analyses was hampered by the impossibility of disentangling these two contributions: chemical oxidation and enzymatic reduction. For instance, the overrepresentation of glutamate is compatible with, at least, two different interpretations: (i) the presence of glutamate close to a methionine residue favors its chemical oxidation to MetO. Alternatively, (ii) it could happen that MetO next to glutamate residues are worse substrates of Msrs. Even worse, the likely formation of secondary oxidants within the living cell [5] adds an additional layer of complexity to the interpretation of the observed results because although H_2_O_2_ acts as a triggering stimulus leading to the formation of MetO residues, we cannot assume that H_2_O_2_ is the main oxidizing agent under such conditions.

The wealth of information provided by the database MetOSite, which allows us to distinguish between MetO sites coming from ex vivo studies and MetO sites formed in vitro, gives us the opportunity to untangle, at least partially, the sequence dependent effects on the oxidation reaction from those on the reduction reaction. In this context, we reasoned in the following way. A differential sequence environment of MetO sites present in living cells may reflect various contributions: presence/absence of amino acids favoring/disfavoring the chemical oxidation reaction as well as the absence/presence of residues favoring/disfavoring the reduction (back) reaction. On the contrary, MetO formed in vitro by treatment with an excess of hydrogen peroxide under conditions incompatible with sustained Msr activity should only reflect the effect of the sequence environment on the oxidation reaction with H_2_O_2_ as the main oxidizing agent.

**Figure 10:**
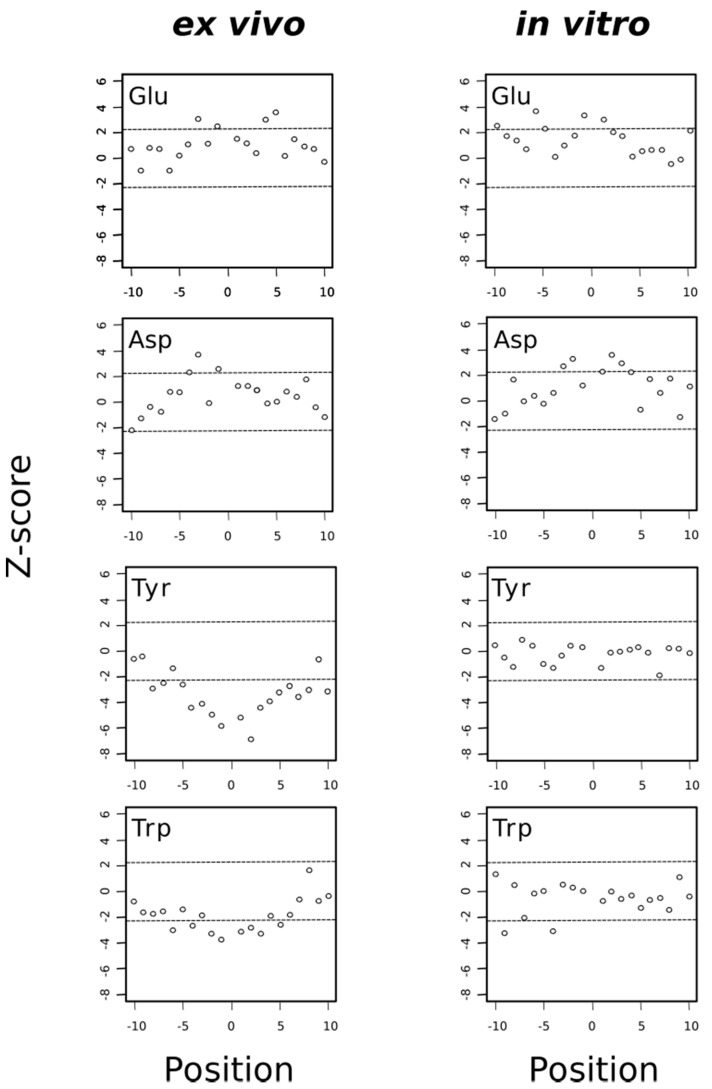
Acidic residues are overrepresented around MetO sites regardless the conditions while aromatic residues are underrepresented only around ex vivo MetO sites. A Z-score that is much greater than zero indicates that the analyzed amino acid is overrepresented at the indicated position. Conversely, a Z-score that is much less than zero indicates that the amino acid being analyzed is underrepresented at that position. The horizontal dashed lines delimit the 99.5% confidence interval of the Z-statistic.

Thus, when we analyzed separately the sequence environments of methionine residues oxidized either ex vivo or in vitro, we found that both acidic amino acids, glutamate and aspartate, tended to be overrepresented in these environments regardless of the experimental conditions (ex vivo or in vitro), suggesting that the presence of acidic amino acids in these regions favored the chemical oxidation of methionine to MetO (Figure 10). Interestingly, it has been described that tyrosine oxidation is enhanced by the presence of neighboring acidic residues [42]. Although the precise role of acidic amino acids in promoting oxidation of target residues is not clear, Bayden and co-workers, using structure-based models, suggested that these acidic residues might play a role by stabilizing intermediate stages where oxidizing agents attack the target residue [43]. Whether this stabilizing effect is also behind the observation that acidic residues are overrepresented in the environment of MetO residues is unknown but in any event our finding that MetO formed in vitro was also situated closer to acidic residues ruled out the explanation that those MetO were poor substrates of Msrs as previously suggested [18]. In fact, the opposite could be true; that is, those MetO next to glutamate and/or aspartate residues may be better substrates of Msrs. As we have discussed above, methionines around acidic residues seemed to be prone to oxidation in different species and under different oxidative stimuli. Therefore, one may expect that the active center of Msrs has evolved to recognize and interact with those negatively charged residues frequently found in the proximity of the target MetO. This seems to be the case at least for MsrP in *Rhodobacter sphaeroides*. Indeed, Tarrago and co-workers have shown that when the periplasmic extracts of this bacterium are treated with NaOCl and then incubated with MsrP, those MetO sites next to glutamate and/or aspartate were more efficiently reduced back to methionine [9].

On the other hand, the aromatic residues tryptophan and particularly tyrosine were clearly avoided in the proximity of MetO originated ex vivo but not in the vicinity of those MetO formed in vitro (Figure 10). As we observed that most proteins oxidized in vitro were cytosolic proteins (Figure 6), we addressed whether the subset of cytosolic MetO sites being originated ex vivo behaved regarding their preferences around the MetO sites similar to the in vitro set of MetO sites. To this end, we carried out a sequence environment analysis restricted to the cytosolic subset of ex vivo oxidized sites, finding that acidic residues were overrepresented while tyrosine and histidine were underrepresented in the MetO environments of the cytosolic subset of protein oxidized ex vivo (Figure S2). Therefore, the avoidance of tyrosine and histidine around MetO sites was mostly to do with the reaction conditions than with the subcellular origin of the protein.

At least three explanations, non-mutually exclusive, could rationalize these findings. One alternative may be that the presence of tyrosine next to MetO increases the efficiency of Msrs, hindering the detection of such sites in ex vivo experiments where Msrs are active but not in in vitro experiments where Msrs are unable to reduce MetO back to Met. A second alternative is that while in in vitro the main oxidizing agent is H_2_O_2_, within living cells the hydrogen peroxide stimulus may lead to the formation of a plethora of secondary oxidants responsible for the oxidation of methionine as well as other vulnerable residues such as tyrosine [44]. In this scenario, methionine and tyrosine may be competing targets of the same oxidizing reagents, which may explain why MetO are detected more often when there are no tyrosines in its proximity. The third and likely more feasible possibility is that within living cells the thioether sulfur of methionine can be oxidized by one-electron oxidants to sulfur radical cation (Met^•+^). This sulfuranyl radical in the absence of a nearby tyrosine can suffer a second one-electron oxidation to yield methionine sulfoxide [5]. However, whenever a tyrosine residue is close enough to the sulfuranyl cation, an intramolecular redox reaction can take place yielding back methionine and leaving behind a tyrosyl radical. Indeed, such radical transitions from methionyl to tyrosyl have been reported to happen in peptides and proteins [45,46].

**Figure 11:**
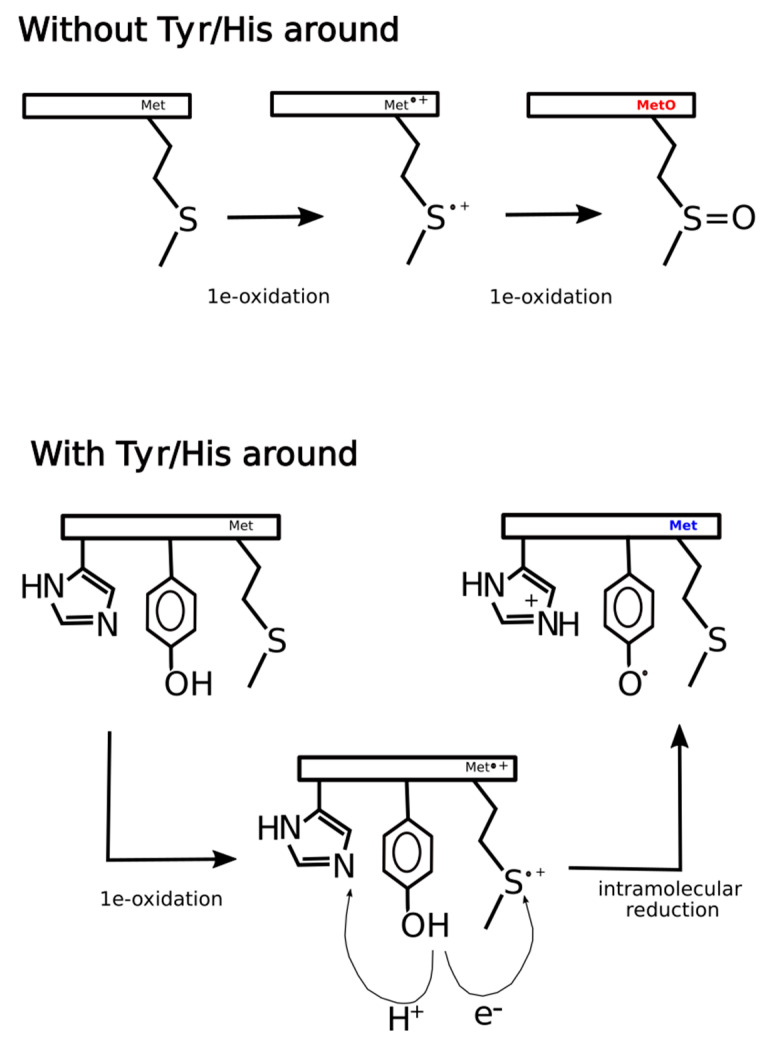
Hypothetical mechanism of ex vivo methionine oxidation. In the absence of tyrosine and histidine in the neighborhood of a given methionine residue, two consecutive one-electron oxidations lead to the formation of methionine sulfoxide. However, if tyrosine and histidine are present in the environment of the methionine site, after the first one-electron oxidation, the intermediate sulfur radical cation can be reduced back to methionine through an intramolecular redox reaction where tyrosine acts as a reducing agent leaving behind a tyrosyl radical. As tyrosine oxidation implicates a concerted deprotonation/oxidation of the hydroxyl group, the transfer of a proton to a nearby proton-accepting histidine residue may facilitate the global reaction.

**Figure 12:**
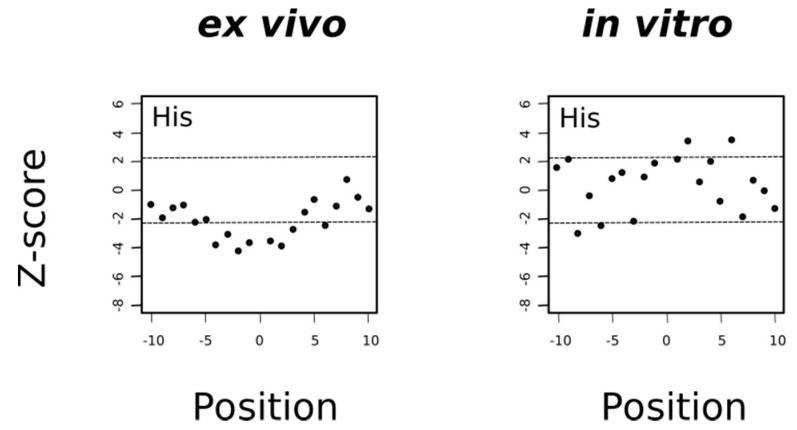
Histidine is avoided in the environment of ex vivo but not in vitro oxidized methionines. Under the null hypothesis conditions (there are no differences in the frequencies of histidine around Met and around MetO), the mean of the Z-statistic must be zero. The horizontal dashed lines delimit the 99.5% confidence interval of the Z-statistic. The filled circles represent the computed Z-score at the indicated position.

In general, the mechanism of tyrosine oxidation implicates concerted deprotonation/oxidation of the hydroxyl group [47]. That is, a concerted transfer of a H^+^ to a nearby proton-accepting base and of an e^-^ to the close oxidizing agent (the sulfuranyl radical) may be the way to reduce Met^•+^ back to methionine. Work on both small molecule and macromolecular systems, reviewed in [47], indicates that a base near the phenolic proton tunes the Tyr-O^•^/Tyr-OH reduction potential and also keeps the proton near the Tyr-O^•^ radical. Examination of 3D structures shows that enzymes that use tyrosine as redox way station typically place a proton-accepting group, generally a histidine residue, near the phenolic proton, thereby ensuring that both H+ and e- are available to reactions [47]. Therefore, if the absence of tyrosine residues around MetO in proteins from the ex vivo set was a consequence of an oxidation/reduction mechanism such as the one we have proposed above and schematized in Figure 11, then we would expect to find also an underrepresentation of histidine residues in the sequence environments of such MetO sites but not in their in vitro counterparts. Indeed, that was what we observed (Figure 12).

## 4. Conclusions

Methionine residues in proteins can be oxidized by reactive oxygen or nitrogen species to generate methionine sulfoxide. This covalent modification has been implicated in processes ranging from normal cell signaling to a wide variety of diseases. However, due to the lack of an adequate methodology, the characterization of the reaction mechanism of methionine oxidation in vivo has been elusive. To overcome this handicap, we developed a computational approach aimed to characterize the set of proteins and methionine sites from the human proteome that may be signaling-competent. Herein we report that those proteins liable to methionine oxidation, either ex vivo or in vitro, are enriched in methionine residues and intrinsically disordered regions. However, these two groups of proteins differ in a number of features. Thus, proteins being oxidized within living cells tend to be larger and less abundant than those proteins reactive in vitro. Regarding the subcellular distribution of these two groups, in vitro oxidized proteins are mainly cytosolic proteins whereas nuclear and mitochondrial proteins are frequently found in the set of proteins oxidized ex vivo. To further investigate the distinctive characteristics of proteins oxidized inside a living cell with respect to those oxidized in vitro, we constructed a protein network based on gene ontology terms. Thus, two given proteins were connected if they shared at least 50% of their gene ontology terms. When this network was analyzed, a very significant (*p*-value < 10^−4^) assortative mixing of these two groups of proteins was found, which indicated that proteins oxidized ex vivo tend to share among them features related to their molecular functions and the biological processes they are involved in; features that are not shared by the proteins belonging to the in vitro set. This conclusion is also supported by the observation that proteins from the ex vivo set were found to be frequently co-regulated in response to diverse stimuli.

When we focus our interest on the sequence environments of MetO sites, a few conclusions can be drawn: (i) regardless of whether the oxidation reaction takes place in vitro or ex vivo, MetO sites are more likely to be found in the neighborhood of the acidic amino acids glutamate and aspartate. (ii) The aromatic residues tyrosine and tryptophan as well as the amino acid histidine are avoided in the proximity of MetO sites only when the methionine has been oxidized within a living cell but not in the case of residues oxidized in vitro. These observations lead us to formulate a working hypothesis: methionine residues in vivo are mainly sulfoxidized following a two elementary steps reaction mechanism where each elementary step consists of a one-electron oxidation process. Further work will be required to determine whether or not this hypothesis is correct.

## Figures and Tables

**Table 1:** Subcellular compartments analyzed and their corresponding gene ontology (GO) terms.

Subcellular Compartment	GO Cellular Component Term	Abbreviation
Mitochondrion	GO:0005739	Mit
Nucleus	GO:0005634	Nuc
Lysosome	GO:0005764	Lys
Endoplasmic reticulum	GO:0005783	ER
Plasma membrane	GO:0005886	Plm
Golgi apparatus	GO:0005794	Gol
Endosome	GO:0005768	End
Cytoskeleton	GO:0005856	Ske
Peroxisome	GO:0005777	Per
Cytosol	GO:0005829	Cyt

**Table 2:** Contingency table for the cluster “Nucleus”.

	Ex Vivo	In Vitro	
Nucleus	255	29	284
Others	858	474	1332
	1113	503	1616

## Supplementary Materials

The following are available online at https://www.mdpi.com/2076-3921/9/10/987/s1, Figure S1: Overrepresentation of acidic residues in the sequence environment of MetO oxidized in vivo in *Arabidopsis thaliana*. Figure S2: Sequence environment analyses within the cytosolic subset of ex vivo oxidized proteins. Table S1: References related to the proteomic studies describing the MetO sites used in the current work. Table S2: Subcellular clusters containing at least 10 proteins from the dataset. Code and raw data can also be obtained at https://bitbucket.org/jcaledo/characterization-of-human-meto-sites.
