# Supplementary material for: Susceptibility of Protein Methionine Oxidation in Response to Hydrogen Peroxide Treatment–Ex Vivo versus In Vitro: A Computational Insight
Source: Antioxidants (Basel). 2020 Oct 13;9(10):987. doi: 10.3390/antiox9100987 (PMC7602125; doi:10.3390/antiox9100987)
Supplement: Supplementary file 1 [file antioxidants-09-00987-s001.pdf]

Supplementary Material

Supplementary Figure S1.
Supplementary Figure S2.
Supplementary Table S1.
Supplementary Table S2.

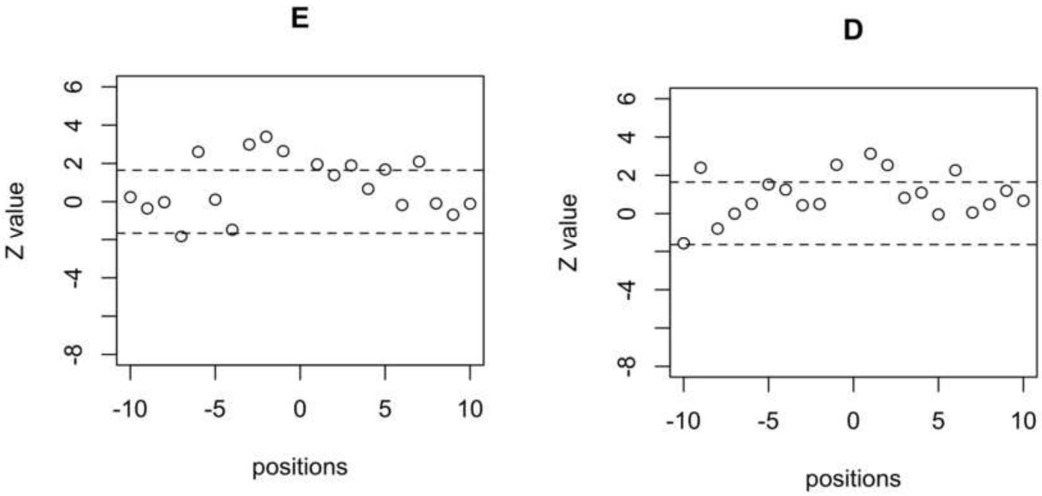

Figure S1. Overrepresentation of acidic residues in the sequence environment of MetO oxidized *in vivo* in *Arabidopsis thaliana*. Data were obtained from MetOSite (https://metosite.uma.es), and they were originated mainly in two proteomic studies (Mol Cell Proteomics 2015, 14(5):1217-29; Cell Commun Signal 2013, 11(1):1).

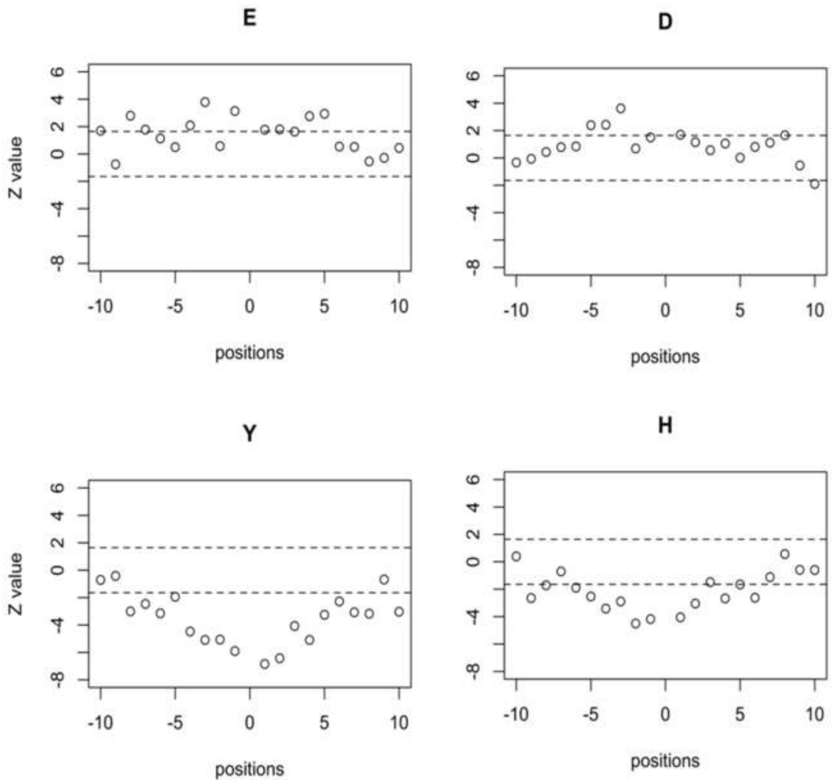

Figure S2. Sequence environment analyses within the cytosolic subset of *ex vivo* oxidized proteins. Acidic residues are overrepresented while tyrosine and histidine are underrepresented in the MetO environments of the cytosolic subset of protein oxidized *ex vivo*.

Table S1. References related to the proteomic studies describing the MetO sites used in the current work. The number of the MetO sites, the title of the paper, the digital object identifier (DOI), the PubMed identifier (PMID) and the dataset to which the sites belong are given. Note that the same MetO site can appear in different studies.

Number of sites	Title	DOI	PMDI	Dataset
2484	Redox proteomics of protein-bound methionine oxidation	10.1074/mcp.M110.006866	21406390	*Ex vivo*
2454	Global analysis of methionine oxidation provides a census of folding stabilities for the human proteome	10.1073/pnas.1819851116	30846556	*In vitro*
26	Quantitative analysis of in vivo methionine oxidation of the human proteome	10.1021/acs.jproteome.9b00505	31801345	*Ex vivo*

Table S2. Subcellular clusters containing at least 10 proteins from the dataset.

a: (% of the cluster proteins contributed by *ex vivo*)

b : (% of the *ex vivo* proteins set contributing to the cluster)

c: Fisher's exact test for count data. those clusters with p-value < 0.02 are emphasized in bold.

Nuc: nucleus; Cyt: cytosol; Mit: mitochondrium; Plm: plasmalemma; ER: endoplasmic reticulum; Ske: cytoskeleton; Gol: Golgi; End: endosome

Cluster	**Compartment**	**Number Proteins**	**% Cluster**[a]	**% Ex vivo set**[b]	**p-Value**[c]
1	***Nuc***	284	89.8	19.2	$< 2\ 10^{-16}$
2	Nuc-Cyt	243	65.0	11.5	0.176
3	***Cyt***	160	44.4	4.9	$1.7\ 10^{-6}$
4	Miscellanea	116	69.9	5.4	0.917
5	***Mit***	80	98.8	5.2	$3\ 10^{-12}$
6	Plm-Cyt	45	57.8	1.7	0.105
7	ER	45	55.6	1.6	0.071
8	Ske-Cyt	43	62.8	1.7	0.405
9	***Nuc-Plm-Cyt***	41	48.8	1.3	0.009
10	Nuc-Ske-Cyt	32	68.8	1.4	0.999
11	Mit-Nuc-Cyt	28	64.3	1.1	0.680
12	Plm	27	66.7	1.1	0.835
13	***Mit-Nuc***	27	92.6	1.6	0.005
14	Mit-Cyt	23	56.5	0.8	0.255
15	***Plm-Ske-Cyt***	22	40.9	0.6	0.009

16	Nuc-Plm-Ske-Cyt	20	50.0	0.6	0.090
17	Gol-Ske	19	68.4	0.8	0.999
18	Ske	16	56.3	0.6	0.285
19	Nuc-Ske	15	73.3	0.7	0.999
20	***Nuc-Plm***	13	100.0	0.8	0.013
21	ER-Cyt	12	66.7	0.5	0.999
22	Plm-Gol-Cyt	12	50.0	0.4	0.208
23	End-Cyt	11	54.5	0.4	0.333
24	Plm-End-Cyt	10	80.0	0.5	0.733
